# Complete Response to Intravesical BCG for High‐Risk pT1 Bladder Cancer With Lymphovascular Invasion: A Case Report

**DOI:** 10.1155/crom/7517129

**Published:** 2026-08-04

**Authors:** Anas Saad, Fahad Mohammad Daraan, Ibrahim Barghouth, Mouhammed Sleiay

**Affiliations:** ^1^ Urology Department, University National Hospital, Damascus University, Damascus, Syria, damascusuniversity.edu.sy; ^2^ Faculty of Medicine, Hama University, Hama, Syria

**Keywords:** BCG therapy, bladder cancer, high-grade NMIBC, pT1 urothelial carcinoma

## Abstract

High‐grade non–muscle‐invasive bladder cancer (NMIBC) with lymphovascular invasion is rare in young adults. We report a 27‐year‐old male, a heavy hookah smoker, who presented with painless gross hematuria. Cystoscopy revealed a large papillary bladder tumor. Transurethral resection demonstrated high‐grade pT1 urothelial carcinoma with lymphovascular invasion and uninvolved muscularis propria. Repeat resection 4 weeks later confirmed no residual malignancy. The patient received six induction doses of intravesical BCG and remains recurrence‐free at follow‐up. This case illustrates an uncommon presentation of high‐risk NMIBC in a young patient and demonstrates an excellent early response to bladder‐preserving therapy despite adverse pathological features.

## 1. Introduction

Bladder cancer is the 10th most common malignancy worldwide and primarily affects patients over the age of 60. Urothelial carcinoma accounts for approximately 90% of all bladder cancers [1]. Non–muscle‐invasive bladder cancer (NMIBC) constitutes nearly 70%–75% of newly diagnosed cases; however, high‐grade T1 disease represents a particularly high‐risk subgroup due to its elevated rates of recurrence and progression to muscle‐invasive disease [2, 3].

The occurrence of high‐grade NMIBC in young adults is rare, and limited data are available regarding its biological behavior and optimal management in this population. Tobacco exposure is a well‐established risk factor for bladder cancer, although the association between heavy hookah smoking and urothelial carcinoma is underreported [3–5].

We present a rare case of a young adult diagnosed with a large high‐grade pT1 urothelial carcinoma with lymphovascular invasion (LVI), successfully managed with transurethral resection and intravesical Bacillus Calmette–Guérin (BCG) therapy.

## 2. Case Presentation

A 27‐year‐old male presented to the urology clinic with painless gross hematuria. He reported heavy daily hookah smoking for several years. There was no history of occupational exposure, urinary tract infections, urolithiasis, chronic medical conditions, or previous surgical procedures.

**Figure 1:**
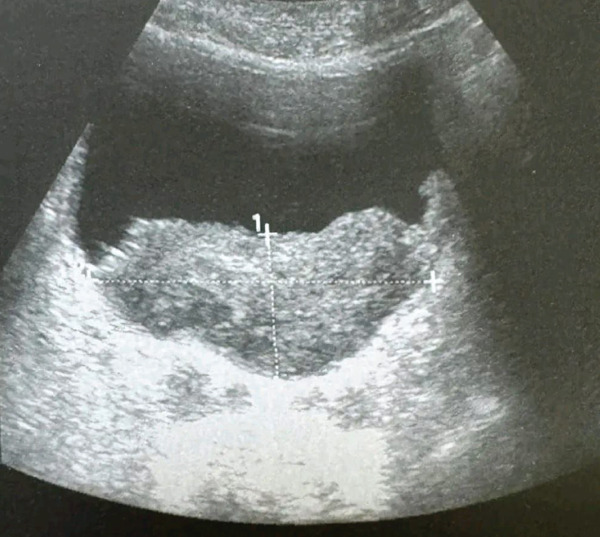
Transabdominal ultrasound of the urinary bladder.

Physical examination was unremarkable. Laboratory investigations, including complete blood count and renal function tests, were within normal limits. Transabdominal ultrasound of the urinary tract was performed as part of the initial diagnostic evaluation. The urinary bladder demonstrated a large, irregular, soft tissue mass. The lesion exhibited heterogeneous echogenicity with surface irregularity and no evidence of posterior acoustic shadowing (Figure 1).

Diagnostic cystoscopy revealed a large, exophytic papillary tumor occupying a substantial portion of the bladder cavity. The tumor appeared friable and vascular, without apparent involvement of the bladder neck or ureteric orifices.

The patient underwent transurethral resection of the bladder tumor (TURBT) with maximal safe resection (Video S1). Deep resection was performed to include the muscularis propria.

**Figure 2:**
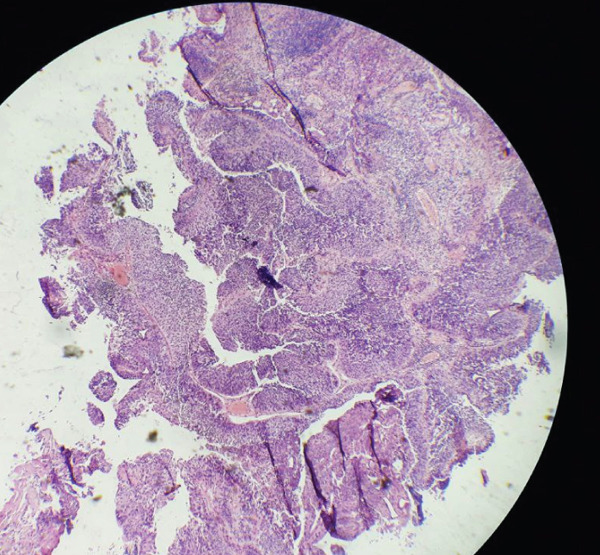
Histopathological examination of the resected tumor (hematoxylin and eosin stain).

**Figure 3:**
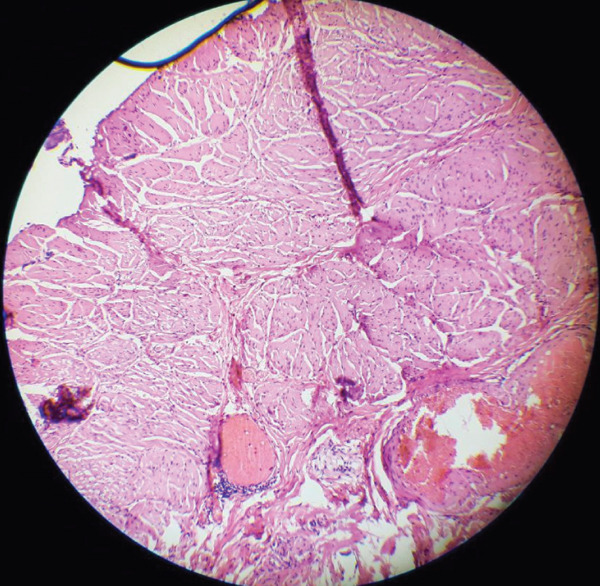
Muscularis propria free of tumor.

Histopathological examination demonstrated high‐grade papillary urothelial carcinoma invading the lamina propria (pT1) (Figure 2). LVI was identified on routine hematoxylin and eosin–stained sections and was confirmed by dedicated review from an experienced genitourinary pathologist. Immunohistochemical staining was not considered necessary, as the morphological features were deemed diagnostic and unequivocal. The muscularis propria was identified and was free of tumor involvement (Figure 3).

Given the high‐risk pathological features, a second‐look cystoscopy with targeted biopsies was performed 4 weeks after the initial TURBT, in accordance with current international guidelines. Cystoscopic evaluation showed a healing resection site without visible tumor. Histopathological analysis revealed ulceration with granulation tissue and nonspecific cystitis, with no evidence of residual malignancy.

The patient subsequently received six weekly induction instillations of intravesical BCG. Treatment was well tolerated without significant adverse effects. Following induction therapy, maintenance BCG was planned according to institutional protocol for high‐risk NMIBC.

The patient has been followed for 12 months after completion of induction BCG therapy. During this period, regular surveillance evaluations, including cystoscopy and urinary cytology, demonstrated no evidence of disease recurrence or progression. The most recent surveillance cystoscopy, performed 12 months after BCG completion, showed a normal bladder mucosa without visible tumor recurrence.

At the most recent follow‐up, the patient remains asymptomatic, reporting no hematuria, irritative urinary symptoms, or obstructive complaints. He continues to demonstrate an excellent response to treatment with no clinical, cystoscopic, or pathological evidence of recurrent disease.

## 3. Discussion

Bladder cancer is predominantly a disease of older adults, with a median age at diagnosis of 73 years and approximately 75% of patients presenting with non–muscle‐invasive disease (NMIBC). In individuals under 40 years of age, bladder cancer is distinctly uncommon, accounting for less than 2% of all cases [1–3]. The present case of a 27‐year‐old male with high‐grade pT1 urothelial carcinoma therefore represents a rare clinical entity that deviates substantially from the typical epidemiological pattern.

Tobacco smoking remains the single most important risk factor for bladder cancer, implicated in approximately 50% of all cases. Carcinogens from tobacco smoke, including aromatic amines and polycyclic aromatic hydrocarbons, undergo renal excretion and accumulate in the urine, resulting in prolonged exposure of the urothelium to carcinogenic metabolites. The risk increases with both duration and intensity of smoking [3, 4]. The patient′s history of heavy daily hookah smoking for several years is particularly noteworthy. Although the carcinogenic potential of waterpipe smoking has been less extensively studied than cigarette smoking, the urine of hookah smokers contains similar carcinogens, and emerging evidence suggests comparable risks for bladder carcinogenesis [2–4].

Recent evidence suggests that young adults with bladder cancer may exhibit distinct clinical and molecular characteristics compared with older populations [2, 3]. A systematic review by Bourgi et al. [5] demonstrated that bladder cancer in young adults is predominantly non–muscle‐invasive and low grade, with generally favorable outcomes. However, this characterization may not fully capture the heterogeneity within this age group. A retrospective study by Agarwal et al. [6] of 162 young bladder cancer patients (≤ 40 years) found that among NMIBC cases, 53.5% had high‐grade tumors, and the disease course could be aggressive, with a mean recurrence‐free survival of 3.7 years. This contradicts the traditional notion that young age uniformly portends indolent disease behavior and underscores the importance of individualized risk assessment.

The diagnostic approach in this case adhered to established guidelines for suspected bladder cancer. Initial presentation with painless gross hematuria in a young male, while more commonly associated with benign etiologies, warranted thorough urological evaluation given the presence of a smoking history. Physical examination and routine laboratory investigations, though unremarkable, are recommended as part of the baseline assessment [7, 8].

Cystoscopy with TURBT remains the gold standard for diagnosis and initial management of bladder cancer. The procedure must accomplish two essential objectives: complete resection of all visible tumor when safely achievable and procurement of adequate tissue for accurate histopathological staging [3–5]. Deep resection to include muscularis propria is critical, as demonstration of muscle invasion (pT2 disease) fundamentally alters prognosis and treatment recommendations. In this case, the muscularis propria was identified and confirmed free of tumor, providing confidence in the pT1 designation [5–7].

The histopathological findings of high‐grade pT1 urothelial carcinoma with LVI classify this patient within the high‐risk NMIBC category according to the European Association of Urology (EAU) risk stratification. LVI is recognized as an adverse prognostic feature associated with increased risk of lymph node metastasis and disease progression. The presence of LVI in a young patient further heightens the clinical concern and typically warrants consideration of more aggressive therapeutic approaches [3, 7, 8].

A critical element in the management of high‐risk NMIBC is performance of repeat transurethral resection (re‐TURBT) within 2–6 weeks of the initial procedure. This recommendation is supported by evidence that residual tumor is identified in 30%–50% of patients with T1 disease at repeat resection, and up to 10% may be upstaged to muscle‐invasive disease. The performance of re‐TURBT at 4 weeks, revealing no residual malignancy, represents optimal adherence to guideline‐directed care and provides important prognostic information. The absence of residual tumor after re‐resection is associated with improved recurrence‐free and progression‐free survival [2–5].

The management of high‐risk NMIBC presents a therapeutic dilemma, balancing the morbidity of radical cystectomy against the oncological risks of bladder preservation. According to current guidelines, patients with high‐grade pT1 tumors, particularly those with associated risk factors such as LVI, large tumor size, or multifocality, should be counseled regarding both radical cystectomy and intravesical BCG therapy as treatment options. The decision‐making process must incorporate patient age, comorbidity status, tumor characteristics, and individual preferences [4–6].

The excellent outcome observed in this case—complete response to induction BCG without recurrence on surveillance cystoscopy—is remarkable given the high‐risk features. Several factors may contribute to understanding this favorable response. First, the complete resection achieved at initial TURBT, confirmed by negative re‐TURBT, provided optimal conditions for intravesical therapy. Second, emerging evidence suggests that young patients may exhibit enhanced responsiveness to BCG immunotherapy. A large Japanese multi‐institutional study by Inoue et al. [9] demonstrated that age ≥ 75 years was an independent prognostic factor for both recurrence and progression following BCG therapy in patients with high‐grade T1 tumors. The cumulative probability of recurrence was significantly higher in older patients, suggesting that younger age may be associated with a more robust immunological response to BCG.

The molecular landscape of bladder cancer in young adults may also differ from that in older populations. Bourgi et al. [5] reported that tumors in younger patients exhibit lower rates of TP53 and FGFR3 mutations compared with those in older adults [3–5]. Although the prognostic implications of these molecular differences require further investigation, they may contribute to the distinct clinical behavior observed in this age group.

Contemporary outcomes data support the approach taken in this case. A recent European multicenter study by Scilipoti et al. [10] comparing upfront radical cystectomy versus BCG therapy in 1491 patients with high‐risk or very high‐risk NMIBC found no significant difference in 5‐year cancer‐specific mortality between treatment groups after propensity score matching (13% for BCG versus 16% for cystectomy [HR: 1.77, 95% CI: 0.66–4.73, *p* = 0.3]). Importantly, among patients who initially received BCG, those requiring delayed cystectomy before disease progression had comparable outcomes with those undergoing upfront cystectomy. However, patients who progressed to muscle‐invasive disease before delayed cystectomy experienced significantly worse cancer‐specific mortality. These findings underscore the importance of close surveillance and timely intervention when indicated.

## 4. Conclusion

This case describes a rare presentation of high‐grade pT1 urothelial carcinoma with LVI in a young adult. Adherence to international guidelines, meticulous surgical technique, and intravesical BCG therapy resulted in an excellent early outcome. This report supports bladder‐preserving strategies in carefully selected high‐risk NMIBC patients.

## Funding

No funding was received for this manuscript.

## Disclosure

The work has been reported in line with the SCARE criteria [11].

## Ethics Statement

The authors have nothing to report.

## Consent

Informed consent was obtained from the patient for the publication of the case details, including publication of clinical images and supporting information.

## Conflicts of Interest

The authors declare no conflicts of interest.

## Supporting Information

Additional supporting information can be found online in the Supporting Information section.

## Supporting information


**Supporting Information 1** SCARE guideline checklist 2025.

**Supporting Information 2** Video S1: Transurethral resection of bladder tumor (TURBT).

## Data Availability

Data sharing is not applicable to this article as no datasets were generated or analyzed during the current study.
